# Genome-Wide Identification of AP2/ERF Transcription Factors in *Capsicum annuum* and Preliminary Functional Analysis of *CaBBM*

**DOI:** 10.3390/plants15152266

**Published:** 2026-07-24

**Authors:** Tong Zhao, Jiayao Wu, Lijun Xian, Yanjie Xiong, Kaiwen Liu, Weiqiang Li, Iqbal Hussain, Xiaolin Yu

**Affiliations:** 1 Department of Horticulture, College of Agriculture and Biotechnology, Zhejiang University, Hangzhou 310058, China; 12216071@zju.edu.cn (T.Z.); 22416239@zju.edu.cn (J.W.); 22516203@zju.edu.cn (L.X.); 22516067@zju.edu.cn (Y.X.); 12016052@zju.edu.cn (K.L.); 12216070@zju.edu.cn (W.L.); iqbal@zju.edu.cn (I.H.); 2 Laboratory of Horticultural Plant Growth & Quality Regulation, Ministry of Agriculture, Hangzhou 310058, China; 3 Group of Vegetable Breeding, Hainan Institute, Zhejiang University, Sanya 572025, China

**Keywords:** AP2/ERF transcription factor family, *Capsicum annuum*, bioinformatics analysis, *CaBBM*, growth development

## Abstract

The AP2/ERF transcription factor family is one of the largest transcription factor families in plants and plays essential roles in growth and development. Chili pepper, as a representative member of the Solanaceae family, is an important vegetable crop with enormous economic value. In this study, using the recently released gap-free telomere-to-telomere genome assembly of pepper, we re-annotated the AP2/ERF transcription factor family and identified 155 high-confidence members. Phylogenetic analysis classified these genes into five subfamilies: AP2 (19), ERF (82), DREB (51), RAV (1), and Soloist (2). Comprehensive analyses of gene structure, conserved motifs, chromosomal distribution, collinearity, *cis*-elements, and expression profiles revealed substantial structural conservation and functional diversification within the family. Expression profiling highlighted *CaBBM*, a key member of the AP2 subfamily, as a candidate developmental regulator, prompting further functional characterization. Expression analyses using qRT-PCR and promoter–GUS assays showed that *CaBBM* was preferentially expressed in stamens, while subcellular localization assays confirmed its nuclear localization. Preliminary analysis of biological functions suggests that heterologous expression of *CaBBM* in *Arabidopsis* can lead to phenotypes such as shorter primary roots, smaller leaves and floral organs, and decreased pollen number. In addition, yeast two-hybrid screening identified 12 candidate interacting proteins. These results provide a comprehensive framework for understanding the AP2/ERF family in chili pepper and lay a foundation for elucidating the function and regulatory mechanisms of *CaBBM*.

## 1. Introduction

The AP2/ERF transcription factor family is one of the largest families of transcription factors in plants and plays diverse roles in regulating plant growth and development. Members of this family are involved in key physiological processes, including seed germination, leaf and root formation, floral organ differentiation, and fruit development [1]. A defining characteristic of the AP2/ERF transcription factor family is the presence of at least one AP2 domain, comprising approximately 60 amino acid residues [2]. Based on the number and structural features of these domains, the family is classified into five subfamilies: AP2 (APETALA2), ERF (Ethylene Reaction factor), DREB (Dehydration reaction element binding factor), RAV (Related to Abscisic Acid Insensitive 3/Viviparous 1), and Soloist [3,4,5].

Among these, the AP2 subfamily regulates key aspects of plant development, including floral organ differentiation, seed and embryo formation, and fruit maturation [6,7]. For example, the *APETALA2* (*AP2*) gene plays a critical role in determining the fate of floral organs during the early stages of flower development, orchestrating their differentiation and proper spatial arrangement [8]. the ectopic expression of *Baby BOOM* (*BBM*), another key member of the AP2 subfamily, has been shown to induce somatic embryogenesis [9]. Notably, in *Brassica napus* and *Solanum lycopersicum*, the ectopic expression of *BBM* in egg cell is sufficient to bypass fertilization and promote independent embryonic development [10]. Similarly, oocyte-specific expression of *OsBBM1* and *OsBBM4* in rice induces parthenogenesis, advancing clonal seed production [11,12]. Moreover, *BBM* genes have been exploited to enhance genetic transformation efficiency [13]. The ERF and DREB subfamilies, which constitute the largest portion of the AP2/ERF family, are primarily involved in mediating plant responses to environmental stresses [14]. ERF proteins are involved in ethylene (ET) signaling and specifically recognize ethylene-responsive elements (EREs) or GCC boxes containing the AGCCGCC core sequence. Overexpression of *RAP2.2* in *Arabidopsis* enhances plant survival under hypoxic conditions, whereas T-DNA insertion mutants exhibit reduced survival compared with the wild type [15]. In contrast, DREB proteins encode dehydration-responsive element-binding factors that interact with dehydration-responsive elements (DREs) or C-repeat (CRT) motifs, characterized by an A/GCCGAC core sequence [1,16]. Notably, recent studies have demonstrated that *OsDREB1C* regulates diverse transcriptional programs influencing photosynthetic efficiency, nitrogen utilization, and flowering time. Its overexpression has been shown to increase rice yield by 41.3–68.3% [17]. The RAV subfamily, while smaller, plays key roles in hormone signaling and mediates plant responses to both biotic and abiotic stresses [18,19,20]. RAV1 in *Arabidopsis* contributes to the regulation of root meristem size and primary root growth by repressing *CRF1* expression and modulating auxin distribution [21]. The Soloist subfamily represents a distinct lineage within the AP2/ERF family, with relatively few members whose biological functions remain largely unexplored [22].

To date, members of the AP2/ERF transcription factor family have been identified in numerous plant species, including model organisms such as *Arabidopsis* [22], rice (*Oryza sativa*) [23], maize (*Zea mays*) [24], tomato (*S*. *lycopersicum*) [25], and tobacco (*Nicotiana tabacum*) [26]. With the rapid advancement of genome sequencing technologies, AP2/ERF genes have also been characterized in a wide range of crop species, including *B. napus* [27], *B. rapa* [28], *Glycine max* [29], and *S*. *melongena* [30]. The AP2/ERF transcription factors exhibit a notable degree of conservation throughout the long-term evolutionary history of plants, while also displaying certain interspecies variations. The number of members within this family varies significantly across different species, a phenomenon that may be influenced by the genome size and the evolutionary divergence of the respective plant species [25]. In pepper, the AP2/ERF transcription factor family was comprehensively characterized by Jin et al. [31], who identified 175 members based on the CM334 genome assembly. While this study established a foundational framework for understanding the AP2/ERF family in *Capsicum* species, the subsequent release of gap-free, telomere-to-telomere (T2T) genome assemblies offers substantially improved genome completeness and annotation accuracy. These enhanced genomic resources provide an opportunity to re-evaluate previously identified gene families, enabling a more precise annotation of AP2/ERF genes and supporting more detailed functional characterization of key family members.

As one of the world’s most economically valuable and widely cultivated crops, chili pepper (*Capsicum annuum*) plays a crucial role in global agriculture. Recent advancements in high-throughput sequencing technologies have greatly accelerated genomic research on chili peppers, leading to significant progress in understanding their genetic makeup. In 2014, the first comprehensive draft of the chili pepper genome was published, marking a major milestone in the field [32]. Recently, the assembly and annotation of two gap-free genomes, *C. annuum* and its wild nonpungent relative *C. rhodoideum*, have been successfully completed [33]. These represent the largest fully sequenced genomes reported to date, providing comprehensive genomic resources for further comparative analysis and functional studies in *Capsicum* species. The investigation of gene families is crucial for understanding the adaptive mechanisms of chili peppers under various environmental pressures, particularly those associated with growth and development, stress tolerance, and disease resistance.

Using the recently released T2T pepper genome assembly, we conducted a genome-wide re-evaluation of the AP2/ERF transcription factor family in *C. annuum*. The identified genes were systematically analyzed in terms of their phylogenetic relationships, gene structures, evolutionary characteristics, and expression patterns. Additionally, we performed an initial investigation into the expression patterns and biological functions of *CaBBM*, a key member of the AP2 subfamily. To further elucidate its potential roles, candidate interacting proteins of CaBBM were screened. These findings provide a foundation for future research into the functional mechanisms of *CaBBM* and its contributions to chili pepper physiology. Collectively, this study offers valuable insights into the functional characterization and regulatory mechanisms of AP2/ERF transcription factors in chili pepper, serving as a key reference for subsequent investigations in this field.

## 2. Results

### 2.1. Identification of AP2/ERF Transcription Factors in C. annuum

Using the recently released T2T genome assembly of chili pepper, AP2/ERF protein sequences from *Arabidopsis thaliana* were employed as query sequences to identify putative AP2/ERF family members through both BLAST (v2.14.0) and hidden Markov model (HMM) searches. The results obtained from the two approaches were integrated, and redundant sequences were removed, yielding 179 candidate genes. Subsequently, conserved domain analysis was performed to validate the presence of AP2 domains, and proteins containing at least one intact AP2 domain were retained, resulting in the identification of 155 putative AP2/ERF family members in chili pepper. Each member was systematically annotated according to its chromosomal position to ensure unambiguous identification. Detailed information for all proteins, including sequence ID, number of amino acids, molecular weight, theoretical pI, instability index, aliphatic index, grand average of hydropathicity, and subcellular localization, is detailed in Table S1. The length of AP2/ERF proteins ranged from 130 amino acids (CaT2T12g03673) to 1190 amino acids (CaT2T01g00260), corresponding to molecular weights of 14.92 kDa and 133.10 kDa, respectively. The predicted pI values varied from 4.39 (CaT2T04g00248 and CaT2T06g03162) to 10.13 (CaT2T01g02171), indicating substantial variation in amino acid composition and physicochemical properties among family members. Only 11 proteins exhibited an instability index below 40, whereas the remaining 144 proteins were predicted to be unstable (instability index > 40), suggesting that most AP2/ERF proteins may possess relatively low intrinsic stability. Such variation in instability index likely reflects differences in protein stability that may be associated with their functional roles and regulatory dynamics. The grand average hydropathicity (GRAVY) ranged from −1.143 (CaT2T03g03204) to −0.228 (CaT2T01g04772), with all values being negative, indicating that all AP2/ERF proteins are hydrophilic. In addition, subcellular localization predictions indicated that these AP2/ERF transcription factors are predominantly localized in the nucleus.

### 2.2. Chromosomal Distribution, Sequence and Structural Analysis of AP2/ERF Transcription Factors

**Figure 1:**
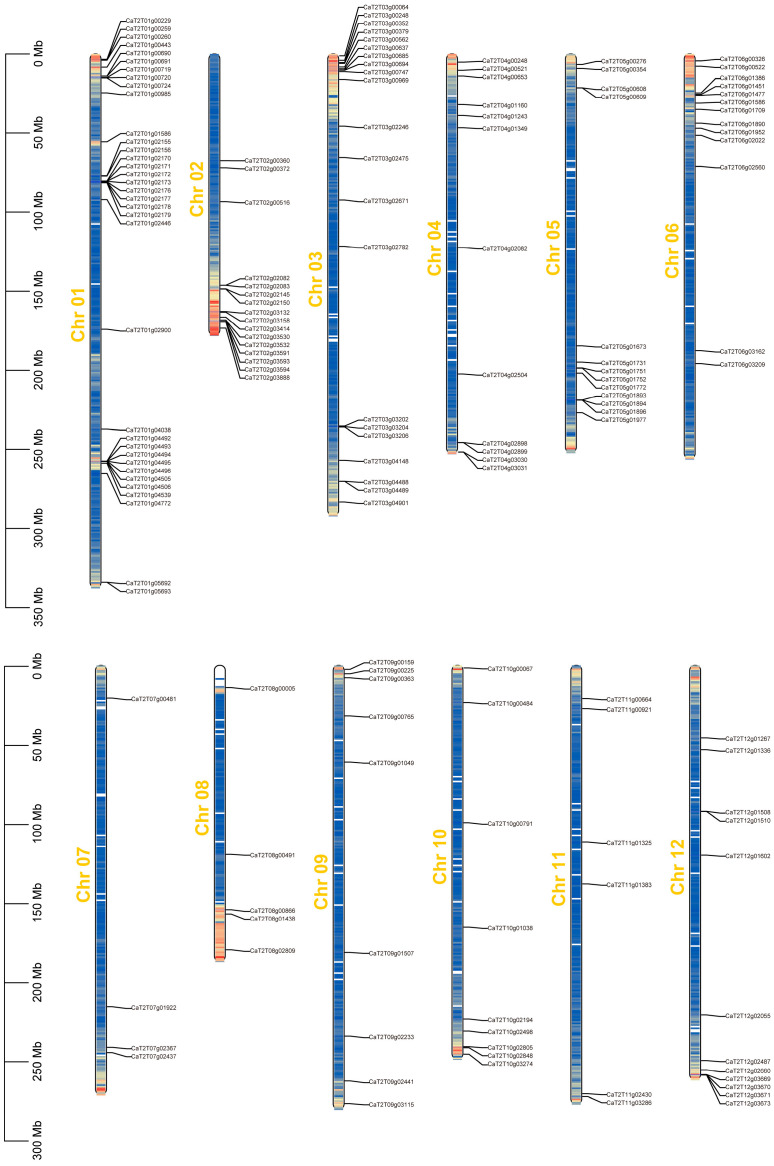
Chromosome distribution of 155 AP2/ERF transcription factors in *C. annuum*. In total, 155 genes are unevenly distributed on 12 chromosomes. The color gradient on chromosomes indicates gene density.

All AP2/ERF transcription factors are unevenly distributed across its 12 chromosomes of *C. annuum*. Chromosome 1 harbors the highest number of members (35), whereas chromosome 7 contains the fewest (4) (Figure 1). To investigate the conservation of these AP2/ERF genes, conserved motifs were analyzed using the MEME Suite (v5.5.9), and gene domains were visualized accordingly. The analysis revealed that most members possessed motifs 1, 2, 3, and 4, while 17 members contained motif 5 and 16 members contained motif 6. Integration of these motif patterns with the phylogenetic tree enabled clear subgroup delineation. Domain analysis indicated that all 155 members contain at least one AP2 domain, one member carries a B3 domain, and 13 members possess two or more AP2-related domains (including AP2 or AP2 superfamily domains). Gene structure analysis indicated that 96 genes were intronless, whereas the remaining 59 genes contained between one and ten introns. Notably, members of the AP2 subfamily exhibited a significantly higher intron content compared with other subfamilies, although two AP2 genes were found to be intronless. Overall, the AP2 subfamily showed an average of 6.16 introns per gene. Collectively, these findings suggest potential functional diversification within the AP2/ERF family (Figure S1).

### 2.3. Phylogenetic Relationship of AP2/ERF Transcription Factors

**Figure 2:**
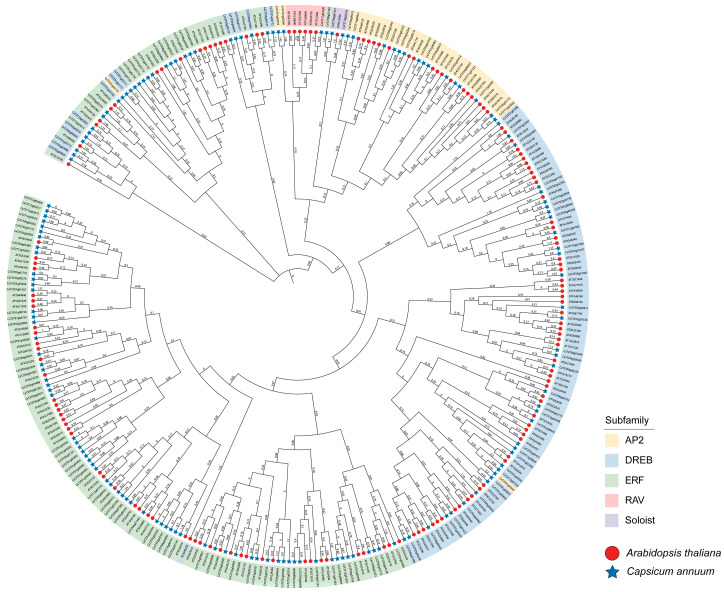
Phylogenetic analysis of AP2/ERF family proteins in chili pepper. The phylogenetic tree was constructed using AP2/ERF proteins from *Arabidopsis* and *C. annuum*. Protein sequences were aligned with MUSCLE, and the phylogenetic tree was inferred using the maximum likelihood (ML) method.

Based on the established classification, the AP2/ERF family is divided into four subfamilies: ERF, AP2, RAV, and Soloist, with the ERF subfamily further subdivided into ERF and DREB groups. To elucidate their evolutionary relationships, a phylogenetic tree incorporating *Arabidopsis* and pepper members was constructed using MEGA X. Although only representative species were included for phylogenetic analysis, this study aimed to focus on the classification and identification of AP2/ERF members in *C. annuum* rather than resolving deep evolutionary relationships within the Solanaceae family. Of the 133 ERF family members, those with alanine (Ala) at position 14 and aspartic acid (Asp) at position 19 of the AP2 domain were assigned to the ERF group, whereas members with valine (Val) at position 14 and glutamic acid (Glu) at position 19 were assigned to the DREB group (Figure S2). Phylogenetic analysis refined this classification, yielding 82 ERF and 51 DREB members (Figure 2).

Thirteen members containing two AP2 domains were designated as typical AP2 proteins, while six single-AP2-domain proteins, phylogenetically closer to the AP2 subfamily than to ERF members, were also included in the AP2 subfamily. A single gene harboring both an AP2 and a B3 domain was classified into the RAV subfamily, and the remaining two genes were assigned to the Soloist subfamily (Figure 2). These results reveal a clear evolutionary partitioning within the AP2/ERF family, reflecting both structural and functional diversification (Table S2).

### 2.4. Synteny Analysis of AP2/ERF Transcription Factors

**Figure 3:**
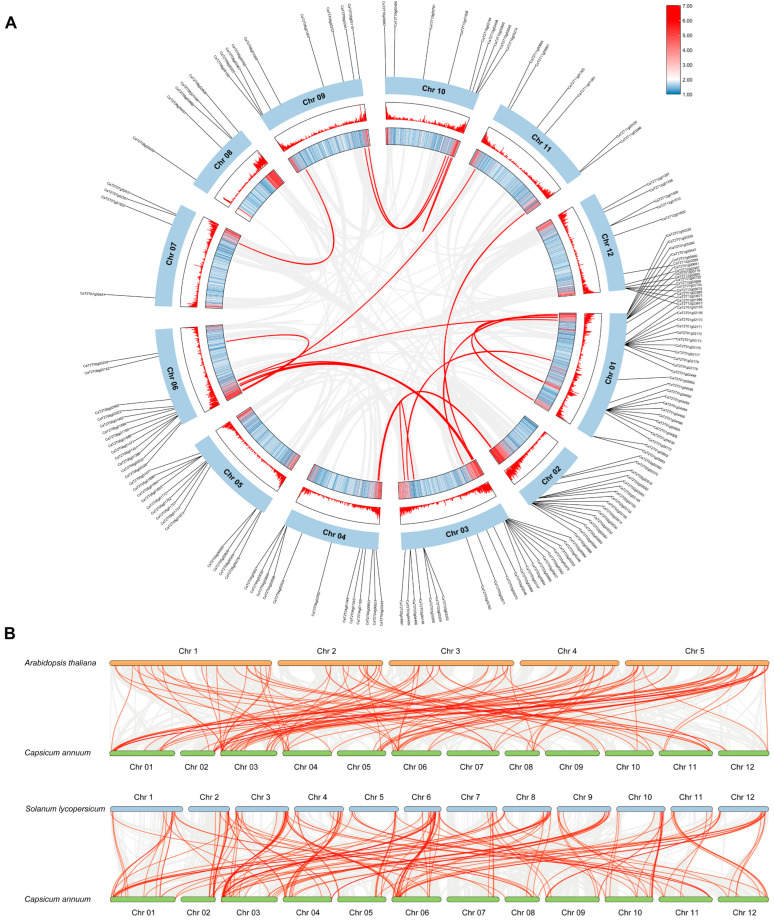
Synteny analysis of AP2/ERF transcription factors in *C. annuum*, *A. thaliana*, and *S. lycopersicum.* (**A**) Duplication events of AP2/ERF transcription factors in *C. annuum*. Red lines indicate duplicated gene pairs, and chromosome numbers are shown within each chromosome. The heatmap represents gene density along each chromosome. (**B**) Intergenomic collinearity of AP2/ERF genes among chili pepper, *Arabidopsis*, and tomato. Grey lines represent genome-wide syntenic gene pairs, whereas red lines highlight the syntenic relationships of AP2/ERF family members among different species.

Intraspecies collinearity analysis of AP2/ERF transcription factors in pepper identified only 22 collinear gene pairs, suggesting limited recent gene duplication events within this family (Figure 3A). Further Ka/Ks analysis revealed that the majority of gene pairs exhibit ratios below 1, indicating that these genes have experienced strong purifying selection (Table S3).

To further explore the evolutionary relationships of the AP2/ERF family, we analyzed collinear gene pairs between chili pepper and two model plants, *Arabidopsis* and tomato. A total of 118 collinear pairs were identified between chili pepper and *Arabidopsis*, whereas 177 pairs were detected between chili pepper and tomato (Figure 3B). These findings indicate a higher degree of collinearity between chili pepper and tomato AP2/ERF genes, reflecting their closer evolutionary relationship and suggesting potential functional conservation between the two species.

### 2.5. cis-Elements Analysis of AP2/ERF Transcription Factors

**Figure 4:**
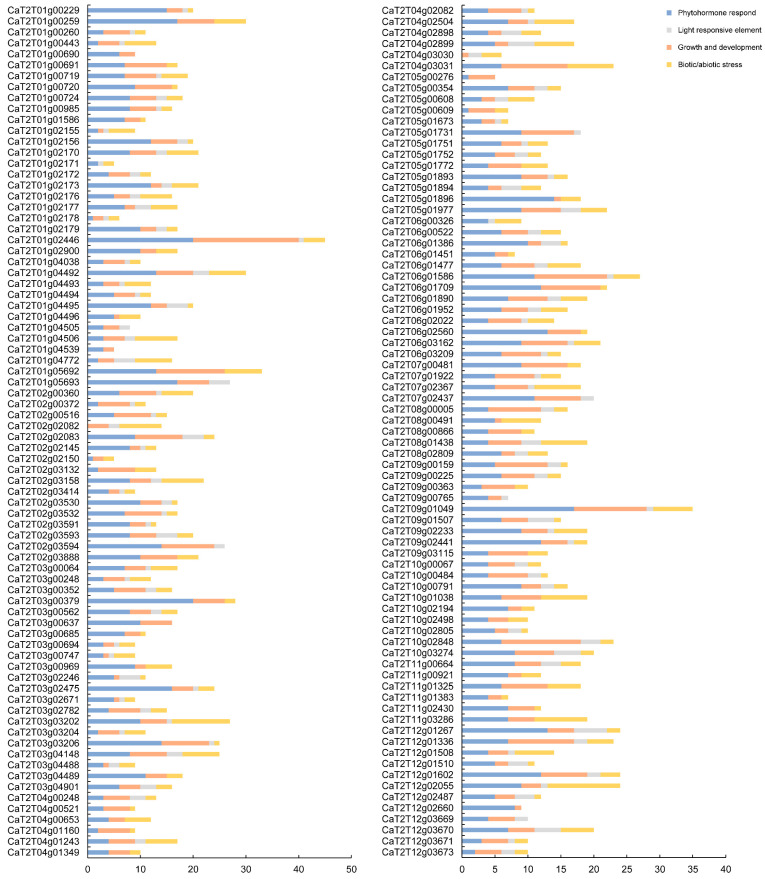
Distribution of *cis*-elements in the promoters of *C. annuum* AP2/ERF transcription factor genes.

A 2000 bp fragment upstream of the start codon of AP2/ERF family genes was extracted as the putative promoter sequence, and *cis*-elements were analyzed using the PlantCARE database. The analysis focused on four categories of *cis*-elements: phytohormone-responsive, light-responsive, growth and development, and biotic/abiotic stress-responsive elements. The results indicated that promoters of AP2/ERF transcription factor genes in pepper contained the highest number of phytohormone-responsive elements, followed by light-responsive and biotic/abiotic stress-related elements, whereas elements associated with growth and development were the least abundant (Figure 4 and Figure S3). Specifically, phytohormone-related elements included motifs responsive to auxin, gibberellin, salicylic acid, abscisic acid (ABA), and methyl jasmonate (MeJA). Growth and development-related elements encompassed motifs involved in cell cycle regulation, circadian rhythm, seed-specific expression, meristem-specific expression, endosperm expression, and endosperm-specific negative regulation. Biotic/abiotic stress-related elements were associated with responses to low temperature, anaerobic conditions, drought, wounding, and other stress factors. Collectively, these results suggest that AP2/ERF family genes in pepper possess diverse regulatory potentials, reflecting their functional versatility in plant growth, development, and stress responses.

### 2.6. Expression Analysis of AP2/ERF Transcription Factors

**Figure 5:**
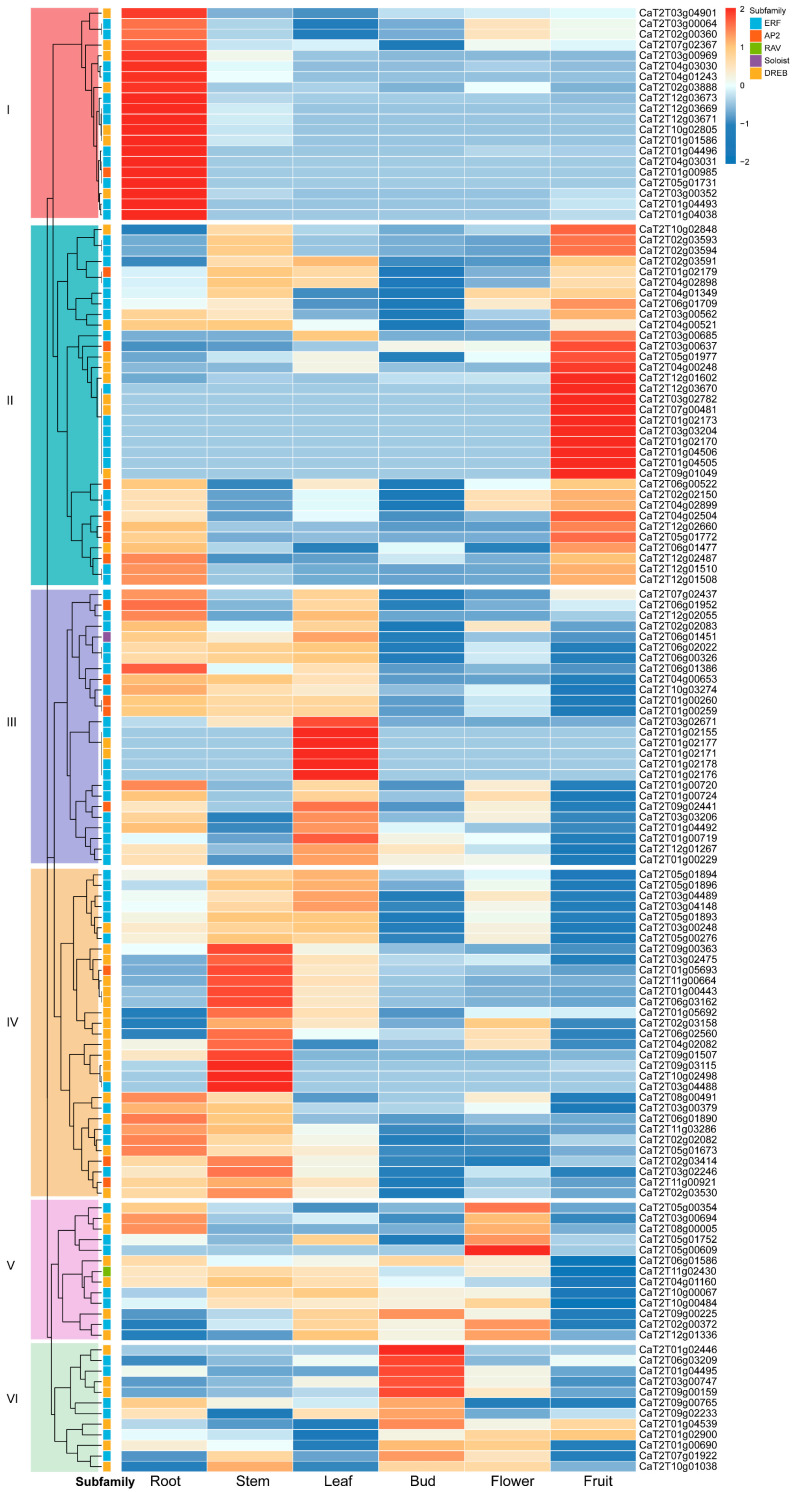
The expression profiles of AP2/ERF transcription factors were analyzed across various tissues, including roots, stems, leaves, flower buds, flowers, and fruits (the ninth developmental stage). In the heatmap, red indicates high expression levels, whereas blue denotes low expression levels. Genes were classified into six clusters (I–VI) according to their expression patterns.

To characterize the tissue-specific expression patterns of AP2/ERF transcription factor family members in chili pepper, expression data for all identified genes were retrieved from PepperBase across roots, stems, leaves, flower buds, flowers, and fruits at the ninth developmental stage. Of the 155 genes analyzed, 12 were absent from the database, and 7 showed no detectable expression in any tissue, leaving 136 genes for further analysis. Hierarchical clustering of these genes based on their expression profiles divided them into six distinct clusters (Figure 5). Cluster I, consisting of 20 genes, exhibited root-specific high expression, while most genes in cluster II were predominantly expressed in fruits. Genes in cluster III show elevated expression in leaves, while the majority of cluster IV genes are highly expressed in stems. Notably, genes in clusters I, III, and IV exhibited strong expression in vegetative tissues—roots, stems, and leaves—while showing lower expression in reproductive organs, including flower buds, flowers, and fruits, suggesting a role in vegetative growth and development. Cluster V genes were primarily expressed in flowers, with a subset exhibiting moderate expression in roots, stems, and leaves. In contrast, genes in cluster VI showed high expression specifically in flower buds. This expression patterns indicate that genes in clusters VI are likely involved in reproductive development, highlighting potential functional specialization among AP2/ERF family members in chili pepper.

To further explore the stress response characteristics of ERF/DREB subfamily members, expression heatmaps were generated using previous transcriptome data under cold, hot, osmotic (mannitol), and salt (NaCl) treatments [34]. Under cold stress, the majority of genes, particularly DREB members, were rapidly induced at the early stage and maintained relatively high expression levels during subsequent treatments, indicating their possible involvement in both early cold response and prolonged adaptation processes (Figure S4A). In contrast, heat stress predominantly triggered rapid and transient responses, with many genes showing strong induction at 12 h followed by gradual downregulation at later stages (Figure S4B). Osmotic stress induced a more pronounced long-term response pattern. Most genes exhibited relatively weak expression changes at 12 h, whereas significant upregulation was observed at 24 h and especially at 72 h, implying potential roles in long-term osmotic adjustment and stress adaptation (Figure S5A). Salt stress displayed both early and late response characteristics. Several genes were rapidly induced at 12 h, while others maintained elevated expression levels at 24 h and 72 h, indicating that NaCl treatment may activate a more complex regulatory network (Figure S5B). Collectively, these results demonstrate that ERF/DREB subfamily genes exhibit distinct temporal and stress-specific expression patterns, highlighting their important regulatory roles in pepper responses and adaptation to abiotic stresses.

**Figure 6:**
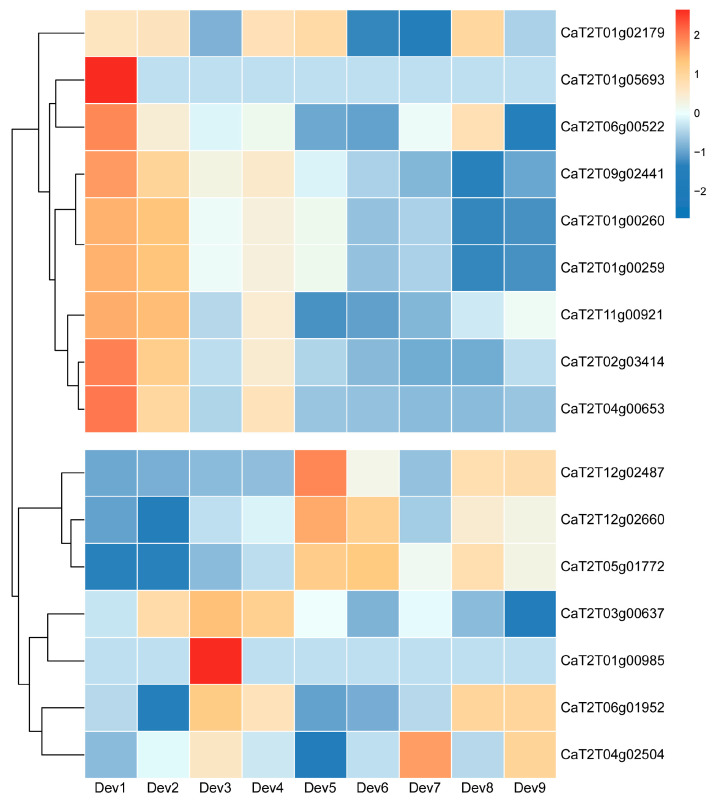
Expression profiles of AP2 subfamily genes across nine stages of fruit development. In the heatmap, red indicates high expression levels, whereas blue denotes low expression levels.

Previous studies have demonstrated that members of the AP2 subfamily play essential roles in multiple developmental processes, including floral organ differentiation, seed and embryo development, and fruit formation. To further investigate the potential roles of AP2 subfamily genes in pepper reproductive development, their expression patterns were analyzed across different fruit developmental stages. Expression data for 16 AP2 subfamily genes were retrieved from PepperBase. Hierarchical clustering analysis revealed two distinct expression patterns: nine genes exhibited relatively high expression during the early stages of fruit development, whereas the remaining seven genes were preferentially expressed during the later stages of fruit development (Figure 6). The presence of two distinct fruit-development expression modules suggests functional specialization among AP2 genes during early and late fruit development.

Among the AP2 subfamily genes, a homolog of the *BABY BOOM* (*BBM*) gene from *Arabidopsis*, designated *CaBBM* (CaT2T12g02487), was selected for further analysis. Among the AP2 subfamily genes, *CaBBM* displayed relatively high expression levels in reproductive tissues and showed marked transcriptional changes during fruit development, suggesting a potential role in development. In addition, *BBM* homologs have been widely reported to play conserved roles in plant regeneration, embryogenesis, and reproductive development [35,36,37,38]. Together, these features suggest that *CaBBM* may act as a key regulatory gene in pepper development.

### 2.7. Expression Analysis of CaBBM in Pepper

**Figure 7:**
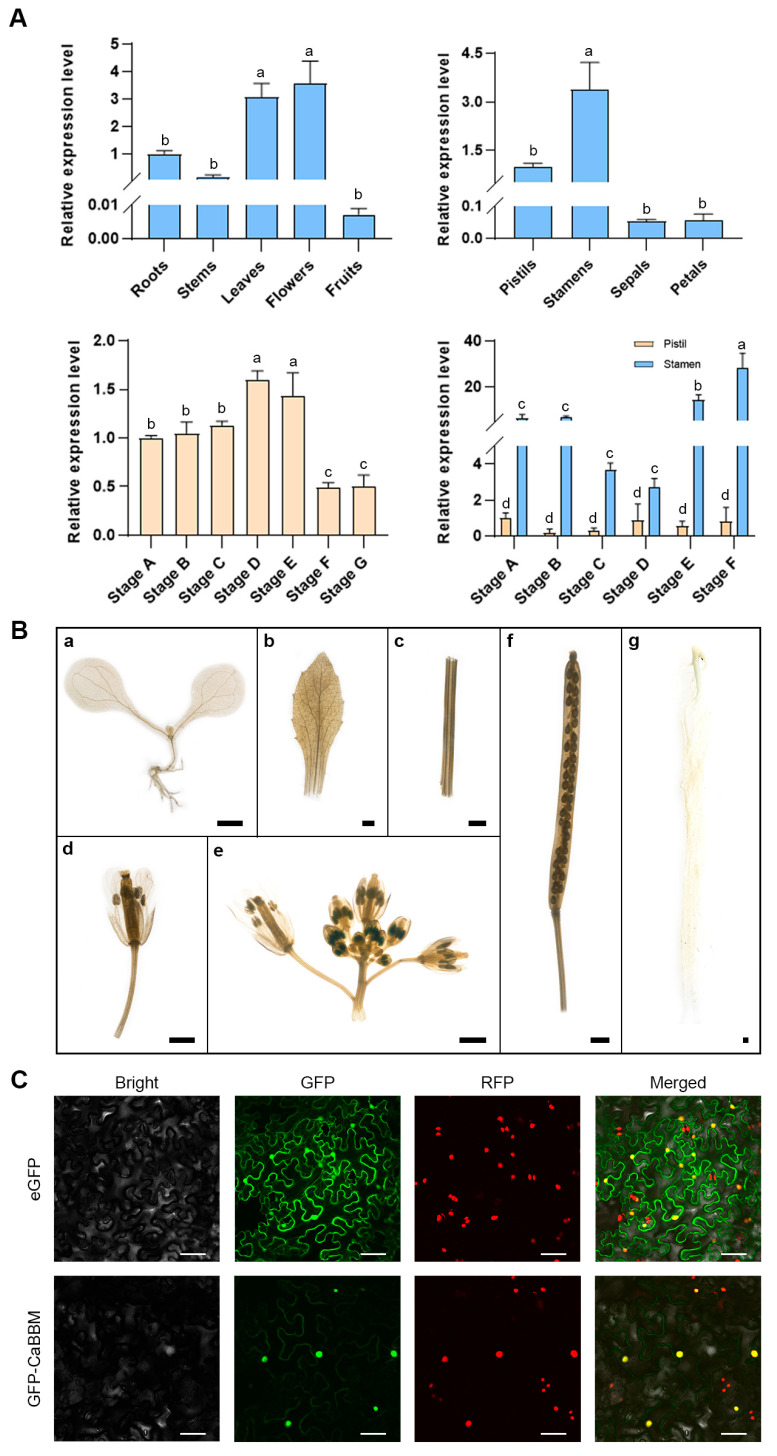
The expression pattern and subcellular localization of *CaBBM*. (**A**) Relative transcript levels of *CaBBM* in different tissues of pepper, as determined by qRT-PCR. Data are presented as mean ± SD, with three biological replicates per group. Statistical significance of relative expression levels among different tissues (roots, stems, leaves, flowers, and fruits), the four floral organs, and seven developmental stages of flower buds were assessed using one-way analysis of variance (ANOVA) followed by Tukey’s multiple comparisons test. Differences in relative expression between pistils and stamens across developmental stages were evaluated using two-way ANOVA followed by Šídák’s multiple comparisons test. Different lowercase letters indicate significant differences among treatments. (**B**) Promoter activity of *proCaBBM::GUS* in *Arabidopsis*. (a) Seedlings; (b) rosette leaf; (c) stem; (d) open flower; (e) inflorescence; (f) silique; (g) the roots of mature plants. Bars, 1 mm. (**C**) Subcellular localization of CaBBM. The nuclear marker H2B–RFP was used to label the nucleus. Bars, 50 μm.

To elucidate the expression pattern of *CaBBM*, its transcript abundance in different tissues of pepper was first examined by qRT-PCR. The results indicated that *CaBBM* is expressed at relatively high levels in floral tissues. To further refine its spatial expression profile, four floral organs were analyzed, revealing that *CaBBM* transcripts accumulate predominantly in the stamens. In addition, flower buds at different developmental stages were assessed, and elevated expression levels were detected at stages D and E. A more detailed comparative analysis of stamens and pistils across multiple bud developmental stages demonstrated that *CaBBM* expression is consistently and significantly higher in stamens than in pistils throughout all examined stages (Figure 7A). These findings suggest that *CaBBM* may play a prominent role in stamen development and function.

To further elucidate the spatiotemporal expression pattern of *CaBBM*, an approximately 2.0 kb upstream genomic fragment was amplified as the putative promoter and used to generate a *proCaBBM::GUS* reporter construct. This construct was subsequently introduced into *Arabidopsis* via the floral dip method, followed by histochemical GUS staining to assess promoter activity. The results showed that no detectable GUS signal was observed in seedlings, leaves, stems, roots, open flowers, or siliques. In contrast, strong GUS activity was specifically detected in the stamens of inflorescences (Figure 7B). This expression pattern is partially inconsistent with the qRT-PCR results, potentially due to limitations in the length of the cloned promoter fragment. Nevertheless, both approaches consistently indicate that *CaBBM* is preferentially expressed in stamens. In addition, the subcellular localization of CaBBM was examined. The protein was found to localize to the nucleus, in agreement with in silico predictions, supporting its putative role as a transcriptional regulator (Figure 7C).

### 2.8. Preliminary Exploration of the Biological Functions of CaBBM

**Figure 8:**
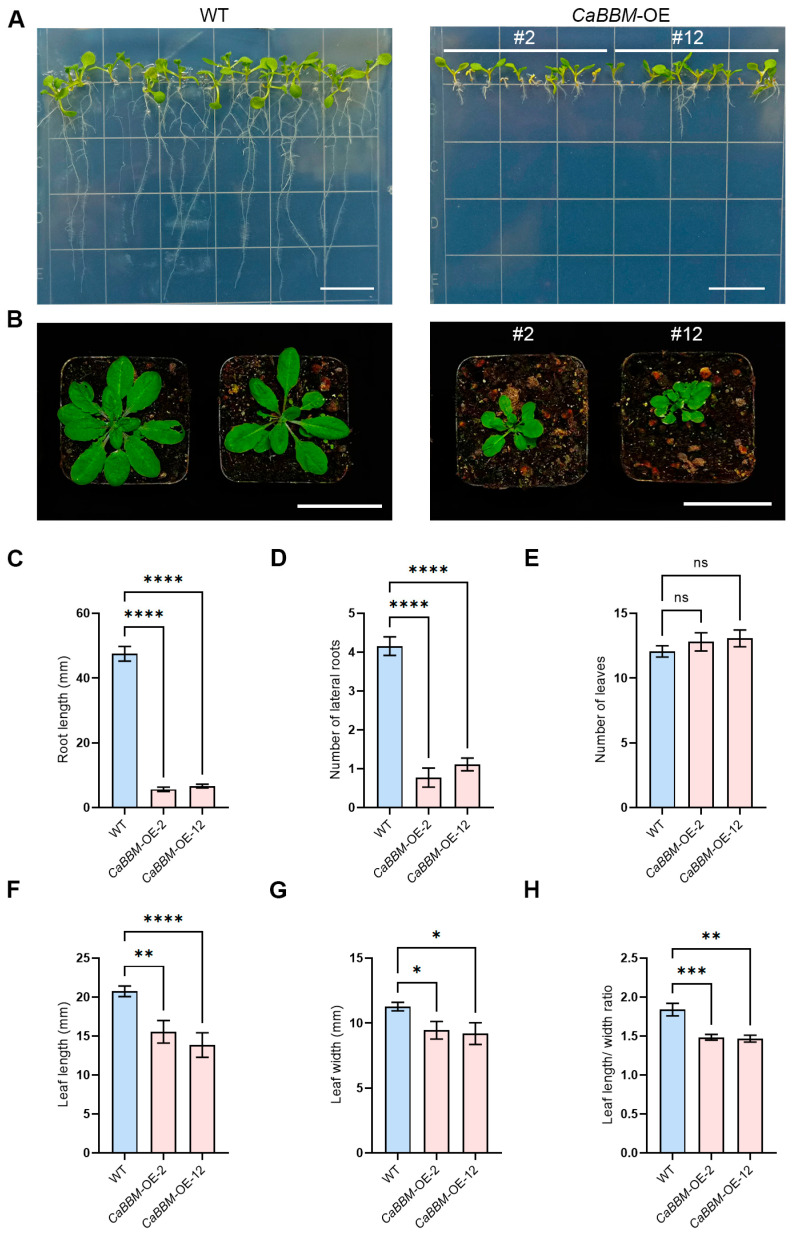
Overexpression of *CaBBM* in *Arabidopsis* affects root and leaf development. (**A**) Root growth of WT- and *CaBBM*-overexpressing plants at 7 days after sowing. Bars, 1.5 cm. (**B**) Growth phenotypes of WT and *CaBBM*-OE plants at 4 weeks after sowing. Bars, 5 cm. (**C**) Quantification of primary root length at 7 days after sowing (*n* ≥ 30). (**D**) Statistical analysis of lateral root number at 7 days after sowing (*n* ≥ 30) (**E**) Total leaf number at 4 weeks after sowing (*n* ≥ 20) (**F**) Leaf length at 4 weeks after sowing (*n* ≥ 20) (**G**) Leaf width at 4 weeks after sowing (*n* ≥ 20) (**H**) Leaf length/width ratio at 4 weeks after sowing (*n* ≥ 20). Data are presented as mean ± SE. Statistical significance was determined using one-way ANOVA and Dunnett’s multiple comparison tests (* *p* < 0.05, ** *p* < 0.01, *** *p* < 0.001, **** *p* < 0.0001; ns, not significant).

Given the considerable challenges associated with the genetic transformation of pepper, the biological functions of *CaBBM* were investigated via heterologous expression in *Arabidopsis*. To this end, a *35S::CaBBM* overexpression construct was generated and introduced into *Arabidopsis* (Figure S6). At 7 days after sowing, *CaBBM*-overexpressing (*CaBBM*-OE) lines exhibited significantly shorter primary roots compared with WT plants, accompanied by a pronounced reduction in lateral root number (Figure 8A,C,D). Phenotypic analyses revealed that heterologous overexpression of *CaBBM* markedly reduced overall plant size (Figure 8B). Leaf-related traits were then quantitatively measured at 4 weeks after sowing. No significant difference in total leaf number was observed between *CaBBM*-OE plants and WT controls (Figure 8E). However, both leaf length and width were significantly decreased in *CaBBM*-OE lines (Figure 8F,G). Moreover, the leaf length-to-width ratio was reduced, indicating a shift in leaf morphology toward a more rounded shape (Figure 8H). Collectively, these findings demonstrate that heterologous overexpression of *CaBBM* exerts a substantial inhibitory effect on vegetative development, particularly with respect to root architecture and leaf morphogenesis.

Floral organs and siliques of *CaBBM* heterologous overexpression lines were subsequently examined. Consistent with the overall reduction in plant stature, floral structures in *CaBBM*-OE plants were uniformly smaller than those of the WT controls, with both pistil and stamen lengths significantly reduced (Figure S7A). In addition, siliques were noticeably shorter in the overexpression lines (Figure S7A). Alexander staining further revealed that, relative to WT plants, *CaBBM*-OE lines exhibited a decreased pollen number, whereas pollen viability was not detectably affected (Figure S7B,C). Quantitative measurements of pollen size using ImageJ (v1.52a) demonstrated that neither pollen diameter nor pollen area differed significantly between *CaBBM*-OE and WT plants (Figure S7D,E). These results suggest that while *CaBBM* overexpression negatively impacts reproductive organ development and pollen production, it does not impair pollen viability or alter pollen morphology.

### 2.9. Screening of Potential Candidate Interacting Proteins for CaBBM

**Figure 9:**
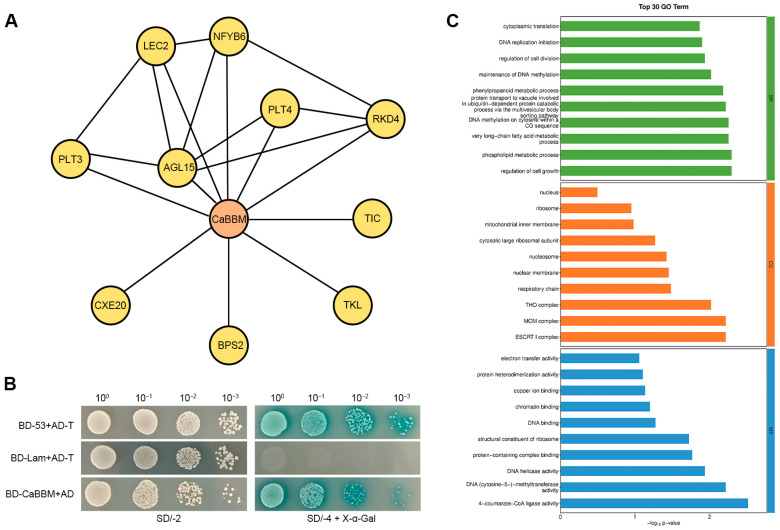
Prediction and screening of CaBBM candidate interacting proteins. (**A**) Predicted PPI network of CaBBM. (**B**) Transcriptional activation activity detection of CaBBM. pGBKT7-53 (BD-53) and pGADT7-T (AD-T) are positive controls, while pGBKT7-Lam (BD-Lam) and pGADT7-T (AD-T) are negative controls. (**C**) GO enrichment results of candidate interacting proteins obtained from yeast library screening.

Protein interaction network analysis was performed using the STRING database (v12.0) based on the *Arabidopsis thaliana* BBM homolog, due to the lack of reliable interaction data for CaBBM. The results suggested that BBM may be functionally associated with several regulators involved in embryogenesis and developmental processes, including LEAFY COTYLEDON2 (LEC2), AGAMOUS-LIKE 15 (AGL15), and Nuclear factor Y B6 (NFYB6) (Figure 9A). These proteins are well known to participate in embryonic development and hormone signaling pathways, implying that BBM may function within a conserved regulatory network governing developmental reprogramming [39,40,41]. Given the evolutionary conservation of BBM proteins, these predicted associations may provide insights into the potential interaction landscape of CaBBM. However, these interactions are based on computational predictions and require further experimental validation.

Subsequently, we used a yeast two hybrid system to verify whether CaBBM has transcriptional activation activity. The results showed that BD-CaBBM + AD can grow on SD/-4 medium, indicating its transcriptional activation activity (Figure 9B). Combined with the previous subcellular localization results, it indicates that CaBBM is a transcription factor located in the nucleus.

In addition, through gradient screening of 3-AT concentrations, we determined that 10 mM is the minimum concentration required to inhibit CaBBM self-activation (Figure S8A), and subsequent yeast library screening identified 12 proteins as potential CaBBM interactors (Figure S8B, Table 1 and Table S4). GO and KEGG enrichment analyses were performed to provide a systems-level overview of potential functional pathways associated with the identified proteins. GO enrichment analysis of the candidate CaBBM-interacting proteins identified by yeast two-hybrid screening revealed significant enrichment in biological processes related to cell growth and division, DNA replication, and epigenetic regulation (Figure 9C). KEGG pathway enrichment analysis further supported these findings, with significant enrichment of pathways associated with DNA replication, consistent with the GO terms linked to cell proliferation (Figure S8C). These results suggest a potential role of CaBBM in modulating cell proliferation, which is consistent with the observed growth inhibition phenotypes in overexpression lines. In addition, several enriched terms were associated with DNA methylation and chromatin organization, including DNA methylation on cytosine within a CG sequence, maintenance of DNA methylation, and nucleosome (Figure 8C). These results suggest that the candidate CaBBM-interacting proteins may be functionally associated with chromatin- and epigenetic-related processes, although these associations are based on enrichment analysis and require further experimental validation. These findings are in line with previous findings that BBM functions in developmental reprogramming and embryogenesis [10].

## 3. Discussion

The AP2/ERF gene family plays critical roles in diverse aspects of plant growth and development and has been extensively characterized across numerous plant species [20]. With the continuous improvement of genome assembly quality, particularly the release of T2T genome assemblies, re-evaluation of previously identified gene families has become increasingly necessary to obtain more accurate and complete annotations. In a previous study based on the genome assembly of the pepper cultivar CM334 (version 2.0), a total of 175 putative CaAP2/ERF genes were identified, comprising 29 members of the AP2 subfamily, 1 member of the RAV subfamily, 144 members of the ERF subfamily, and a single Soloist gene [31]. In contrast, only 155 high-confidence AP2/ERF genes were identified in the present study using the recently released T2T genome assembly of the *C. annuum* doubled haploid line ‘G1-36576’ (Figure 1). Differences in genome assembly quality and annotation strategies can substantially influence gene prediction and gene family identification. Compared with earlier genome versions, the T2T assembly provides a more continuous and accurate representation of the pepper genome, which may help reduce redundant, fragmented, or incorrectly predicted gene models present in previous annotations [33]. In addition, these two genome assemblies are derived from different pepper genotypes, genetic variation among different pepper cultivars may also contribute to differences in AP2/ERF family composition.

The identification pipeline used in this study also applied relatively stringent criteria for family member confirmation. Combined BLAST and HMM searches initially identified 179 candidate AP2/ERF genes, a number comparable to previous reports. However, all candidates were subsequently subjected to conserved domain verification, and only proteins containing at least one canonical AP2 domain (PF00847) were retained. This filtering step reduced the final number of retained genes from 179 to 155 by excluding candidates lacking the canonical PF00847 AP2 domain. Among the 155 AP2/ERF genes identified in this study, 136 (87.7%) showed correspondence with 141 genes reported previously, whereas 19 (12.3%) genes were newly identified in the T2T assembly (Table S6). The difference in gene number likely reflects redundant or fragmented gene models in the earlier annotation, where multiple previously annotated loci corresponded to a single gene model in the T2T assembly. All 19 newly identified genes contained conserved AP2 domains and were therefore classified as AP2/ERF family members. These newly identified AP2/ERF genes may represent previously unresolved loci located in repetitive or structurally complex genomic regions that became accessible through T2T assembly. In contrast, 34 genes previously annotated as AP2/ERF family members could be mapped to the T2T genome by BLAST analysis. To further investigate the discrepancies between the CM334 and T2T genome annotations, we systematically analyzed the 34 AP2/ERF genes that could be mapped to the T2T assembly but lacked a complete PF00847 domain (Table S7). Notably, several CM334 gene models were found to correspond to a single T2T locus, indicating that previous annotations were fragmented. For example, three genes (CaAP2/ERF023, CaAP2/ERF025, and CaAP2/ERF027) were all mapped to CaT2T01g02178, and three additional genes (CaAP2/ERF091, CaAP2/ERF092, and CaAP2/ERF093) were merged into CaT2T04g02897. These results suggest that the improved continuity of the T2T assembly has enabled the consolidation of previously fragmented gene models. In addition to gene structure discrepancies, domain analysis revealed that these genes lacked the canonical AP2 domain (PF00847) and instead exhibited incomplete or inconsistent conserved domain signatures in CDD and SMART databases. This pattern suggests that a subset of these genes may represent false-positive predictions or non-canonical AP2/ERF-like proteins in the previous annotation. Although some weak or partial similarities to AP2-related domains were detected, they were insufficient to meet the criteria for confident AP2/ERF family classification, and therefore these genes were excluded from the final dataset. Despite the difference in total gene number, the overall subfamily composition and phylogenetic classification patterns identified in the present study were largely consistent with previous reports, supporting the reliability and robustness of the current annotation.

The structural features of AP2/ERF proteins provide important insights into their evolutionary conservation and functional diversification. In the present study, conserved motif analysis revealed that most CaAP2/ERF proteins shared several common motifs, particularly motifs 1–4, indicating a high degree of structural conservation within this transcription factor family and these conserved motifs likely represent core functional elements associated with DNA-binding specificity, transcriptional regulation, or protein–protein interactions (Figure S1). However, distinct subfamilies exhibited variability in the conserved motifs. Similarly, four conserved motifs were identified in the AP2 domain of cauliflower (*B*. *oleracea* L. var. *botrytis*), present in nearly all AP2/ERF members, while additional motifs were specific to certain subgroups [42]. In Moso Bamboo (*Phyllostachys edulis*), proteins in subgroups IIb, IIIc, IIId, and VIIa contain LWSY motifs at their C-terminus, while EAR motifs are found in subgroups IIId, IIIc, and VIIIa [43]. These findings collectively suggest that, although the AP2/ERF protein family has undergone evolutionary differentiation, its core structural features have been largely preserved. Gene structure analysis further revealed substantial variation in intron organization among AP2/ERF members, with a notable enrichment of intron-containing genes in the AP2 subfamily. The higher intron density observed in AP2 subfamily members may reflect their involvement in more complex developmental regulatory processes, particularly those associated with floral organ identity and reproductive development. The preservation of complex exon–intron structures may facilitate alternative splicing and transcriptional fine-tuning during reproductive development. Conversely, intronless genes may represent more rapidly responsive transcriptional regulators, particularly within stress-associated ERF/DREB groups.

The intraspecies collinearity analysis of AP2/ERF transcription factors in pepper revealed a relatively small number of collinear gene pairs, with only 22 identified. This suggests a limited occurrence of recent gene duplication events within this transcription factor family in pepper (Figure 3A). The predominance of purifying selection further indicates that AP2/ERF genes are under strong evolutionary constraints, consistent with their central roles in developmental and stress-response regulatory networks. To gain a broader understanding of the evolutionary dynamics of the AP2/ERF family, we extended our analysis to include collinearity between chili pepper and two model plant species, *Arabidopsis* and *S. lycopersicum*. The higher number of collinear gene pairs between pepper and tomato compared to pepper and *Arabidopsis* indicates a closer evolutionary relationship between these two species, which is consistent with their shared Solanaceae lineage (Figure 3B). This finding suggests that AP2/ERF genes in chili pepper and tomato may have conserved similar functions during evolution, further reinforcing the idea of functional conservation across species within this family. Similar results were reported in other studies, which also found a higher degree of gene collinearity between tomato and other Solanaceae species [30,44].

Expression profiling revealed clear tissue-specific and developmental stage-specific patterns among AP2/ERF genes, indicating functional partitioning within the family. Genes preferentially expressed in vegetative tissues likely contribute to root, stem, and leaf development, whereas flower- and fruit-preferential genes are likely involved in reproductive development (Figure 5). Notably, a subset of AP2 subfamily genes showed strong expression during fruit development, suggesting potential roles in reproductive organ formation and maturation processes (Figure 6). The identification of two distinct expression modules among AP2 genes during fruit development suggests functional specialization within this subfamily. Genes preferentially expressed during early fruit development may participate in cell division and organ initiation, whereas late-expressed genes may contribute to fruit maturation and tissue differentiation.

Distinct expression dynamics were observed among ERF/DREB members under different stress conditions, reflecting functional diversification within this subfamily. Many DREB genes showed rapid and sustained induction under cold treatment, consistent with their established roles in activating cold-responsive pathways and promoting acclimation to low temperatures [45]. By contrast, heat stress generally triggered transient expression changes, suggesting involvement in early stress signaling [46]. Osmotic stress induced a delayed but persistent transcriptional response, suggesting that a subset of ERF/DREB genes may be involved in long-term physiological adjustment processes, such as osmotic balance maintenance and metabolic reprogramming. Salt stress triggered both early and late responsive genes, indicating that salinity tolerance may require coordinated regulation of multiple signaling pathways associated with ionic homeostasis, osmotic stress, and reactive oxygen species detoxification. Together, these findings highlight the functional diversification of ERF/DREB family members in pepper and suggest that different subsets of genes may contribute to stress adaptation through distinct temporal regulatory mechanisms [47].

The genome-wide identification and expression profiling of AP2/ERF genes provided a framework for prioritizing candidate regulators of pepper development. Among them, *CaBBM* emerged as a promising candidate because of its developmental expression pattern and the conserved functions of *BBM* homologs in other species. In this study, we characterized the expression pattern and preliminary biological functions of *CaBBM* in pepper. Our qRT-PCR and GUS reporter analyses consistently demonstrated that *CaBBM* is preferentially expressed in stamens, suggesting that *CaBBM* is associated with reproductive organ development in pepper (Figure 7A,B). Previous studies have reported that *Arabidopsis BBM* is predominantly expressed during embryogenesis and seed development, whereas *OsBBM1* in rice is specifically expressed in germ cells [9,11]. The stamen-biased expression observed here is consistent with the germline-associated expression pattern reported in rice. This divergence may indicate species-specific recruitment of *BBM* genes into reproductive developmental programs. Heterologous overexpression of *CaBBM* in *Arabidopsis* resulted in significant inhibition of growth, including reduced root elongation, fewer lateral roots, smaller leaves with altered morphology and pollen reduction (Figure 7 and Figure S7). These observations are in agreement with previous studies showing that ectopic expression of *BBM* markedly influences plant growth and development, producing diverse phenotypic outcomes. Some plants exhibit enhanced growth, whereas others display reduced size, slow vegetative growth, overproliferation of rosette leaves, and delayed flowering, yet remain fully fertile [9]. Notably, overexpression did not induce ectopic somatic embryogenesis, indicating that *BBM* function may differ among species. These findings provide a preliminary insight into *CaBBM* function in chili pepper, although heterologous expression cannot fully recapitulate its intrinsic role because the regulatory networks, downstream targets, and developmental contexts may differ between *Arabidopsis* and pepper. Such discrepancies between endogenous expression patterns and heterologous overexpression phenotypes may result from dosage imbalance and ectopic expression effects commonly observed in *BBM* family genes. Future studies employing functional analyses in pepper, such as transient overexpression, gene silencing, or stable transformation, will be required to validate the native biological functions and regulatory mechanisms of *CaBBM*.

Protein interaction prediction based on *Arabidopsis* BBM homologs suggested potential associations with key developmental regulators such as LEC2, AGL15, and NFYB6, all of which are involved in embryogenesis and transcriptional regulation (Figure 8A). Notably, LEC2 and AGL15 are well-established regulators of somatic embryogenesis and developmental transitions [48,49]. These observations suggest that components of the BBM-associated regulatory network may be partially conserved across species; however, the interaction partners identified in pepper appear to differ from those predicted based on *Arabidopsis* homologs. This discrepancy may reflect species-specific differences in BBM-centered regulatory networks between *Arabidopsis* and pepper. Several candidate interacting proteins were associated with DNA methylation and chromatin-related functions, suggesting a possible link between CaBBM and epigenetic regulatory processes (Figure 8C). However, such associations are based on computational enrichment analyses and these interactions require experimental validation, such as BiFC, Co-IP, or pull-down assays. In addition to chromatin-associated proteins, several candidate interactors were annotated as heavy metal-binding proteins. Heavy metal-associated proteins have been implicated in maintaining cellular metal homeostasis and oxidative stress responses. Because members of the AP2/ERF family are widely involved in abiotic stress responses, these proteins may represent potential candidates for further investigation. Alternatively, some candidates identified by yeast two-hybrid screening may represent false-positive interactions, which is an inherent limitation of library-based screening and requires independent validation. CaBBM also possesses transcriptional activation activity and is localized to the nucleus, consistent with its function as a transcription factor. Together, these findings provide preliminary insights into the regulatory network of CaBBM, highlighting downstream targets that warrant further investigation.

Increasing evidence indicates that *BBM* genes play pivotal roles in enhancing plant transformation efficiency. For instance, ectopic expression of *BnBBM* improves transformation in pepper [50], while *MdBBM1* enhances regeneration in apple [51]. In maize, co-expression of *BBM* and *WUS2* with PMI increases transformation efficiency and shortens the cycle in the inbred line Jing724 [52]. Beyond transformation, *BBM* has also been implicated in parthenogenesis induction. *OsBBM1* triggers haploid embryo formation and, when combined with meiosis-related gene editing, enables synthetic apomixis with high clonal propagation efficiency [11,53,54], whereas *OsBBM4* induces parthenogenesis with lower efficiency [12]. Given the recalcitrance of chili pepper to genetic transformation, the role of *CaBBM* in inducing parthenogenesis and enhancing transformation efficiency could not be examined in stable transgenic lines. Future studies are needed to further elucidate its functions in greater depth and breadth. Our findings provide a foundation for further functional characterization of *CaBBM*.

## 4. Materials and Methods

### 4.1. Identification and Phylogenetics of AP2/ERF Transcription Factors in C. annuum

The gap-free, T2T genome assembly of *C. annuum* was obtained from the Capsicum Genome Database (PGDB; http://www.bioinformaticslab.cn/PepperBase/, accessed on 20 December 2025). BLASTP searches were performed using *Arabidopsis* AP2/ERF protein sequences as queries against the *C. annuum* proteome with an E-value threshold of 1 × 10^−5^. Hits were retained with sequence similarity ≥ 30% and alignment coverage ≥ 50%. In parallel, the hidden Markov model (HMM) profile of the AP2/ERF domain (PF00847) was obtained from the Pfam database and used to search the pepper proteome using HMMER 3.0’s hmm search, applying the same E-value cutoff of 1 × 10^−5^. Candidate sequences identified by both methods were compared, and only overlapping sequences were retained after removing duplicates. For genes with multiple transcript isoforms, only the longest isoform was retained as the representative sequence to avoid redundancy. Finally, all candidate proteins were subjected to domain verification using NCBI’s Conserved Domain Database (CDD). Only proteins containing at least one AP2 domain (PF00847) with an E-value < 1 × 10^−5^ in Pfam or confirmed by NCBI CDD (E-value < 0.01) were retained. The AP2 domain sequences of the identified CaAP2/ERF proteins were extracted and aligned using ClustalW in MEGA X, with alignment results visualized in ESPript 3.x.

Based on protein domain composition, all AP2/ERF proteins were classified into their respective subfamilies. Phylogenetic analysis of AP2/ERF proteins from two species was performed using MEGA X. Protein sequences were aligned using MUSCLE, and a maximum likelihood (ML) phylogenetic tree was subsequently constructed with 1000 bootstrap replicates using the Jones–Taylor–Thornton (JTT) amino acid substitution model. The JTT substitution model was used as it is widely applied in protein-based phylogenetic analyses of plant transcription factor families, including AP2/ERF proteins. All remaining parameters were set to their default values. The resulting phylogenetic tree was visualized and refined using the iTOL (v7) and Adobe Illustrator 2023.

The basic physicochemical properties of the AP2/ERF family members, including protein length, molecular weight (kDa), isoelectric point (pI), and hydropathicity, were analyzed using TBtools (v2.446) [55].

### 4.2. Chromosomal Distribution, Conserved Motif, and Conserved Domain Analysis

The chromosomal distribution of AP2/ERF family members in *C. annuum* was visualized using TBtools. Conserved domains were identified using the Batch CD-Search tool (https://www.ncbi.nlm.nih.gov/Structure/bwrpsb/bwrpsb.cgi, accessed on 22 December 2025), and conserved motifs were analyzed using MEME Suite (https://meme-suite.org/meme/, accessed on 28 December 2025) with the following parameters: maximum number of motifs set to 10, and motif width ranging from 6 to 50 amino acids, using the default distribution model (zoops). Protein sequences were aligned in MEGA X, and a ML phylogenetic tree was constructed. The methods for sequence alignment and phylogenetic tree construction are consistent with standard procedures described previously. Finally, the results were integrated and visualized using TBtools.

### 4.3. Collinearity Analysis

Collinearity within *C. annuum* and between *C. annuum* and *Arabidopsis thaliana* or *Solanum lycopersicum* was analyzed and visualized using TBtools. Synteny analysis was conducted using the Python implementation of MCScan in the JCVI package. Gene coordinate files were generated from the genome annotation (GFF3) file using jcvi.formats.gff bed --type=gene, with only the longest transcript retained for each locus. Homologous gene pairs were identified using jcvi.compara.catalog ortholog with the parameters --no_strip_names and --cscore 0.99. Syntenic blocks were filtered using jcvi.compara.synteny screen (--minspan 30 --simple), and only blocks containing at least 30 anchor pairs were retained. Dot plots and chromosome-level synteny maps were generated using the JCVI graphics module.

### 4.4. cis-Elements Analysis

A 2000 bp sequence upstream of the start codon of each *C. annuum* AP2/ERF gene was extracted as the putative promoter region. *Cis*-elements were identified using the PlantCARE (https://bioinformatics.psb.ugent.be/webtools/plantcare/html/, accessed on 23 January 2026) [56], and the results were visualized with TBtools.

### 4.5. Gene Expression Analysis Based on Transcriptome Data

Gene expression data of AP2/ERF family members across different tissues of *C*. *annuum* were retrieved from the PepperBase (http://www.bioinformaticslab.cn/PepperBase/, accessed on 23 May 2026) database. In addition, transcriptome data from Kang et al. were used to analyze AP2/ERF gene expression under various stress conditions [34]. Expression values were calculated as TPM (Transcripts Per Million). For comparative analysis, TPM values were transformed using log2 (TPM + 1) normalization. For hierarchical clustering and heatmap visualization, Z-score normalization was further applied to the expression matrix to standardize expression levels across genes. All expression analyses were performed using normalized and processed datasets as described above.

### 4.6. Plant Materials and Growth Conditions

Pepper plants of the cultivar ‘Hangjiao-9’ were obtained from Hangzhou Sanjiang Seed Industry Co., Ltd. (Hangzhou, China) and cultivated in greenhouse conditions at Zhejiang University (Hangzhou, China). *Nicotiana benthamiana* and *Arabidopsis thaliana* (Col-0) were grown in controlled growth chambers under a 16 h light/8 h dark photoperiod, with temperatures maintained at 25 ± 1 °C during the light phase and 20 ± 1 °C during the dark phase.

### 4.7. RNA Extraction and qRT-PCR

Tissues were harvested from various organs of pepper, including roots, stems, leaves, flowers, fruits, floral whorls, flower buds at different developmental stages, and pistils, for subsequent analyses. Flower bud staging was performed following the criteria described by Zhao et al. [57]. Total RNA was isolated using the RNA isolater Total RNA Extraction Reagent (Vazyme, Nanjing, China), and first-strand cDNA was synthesized with the HiScript IV All-in-One Ultra RT SuperMix for qPCR (Vazyme). qRT-PCR was carried out using SupRealQ Purple Universal SYBR qPCR Master Mix (U+) (Vazyme) on a Bio-Rad CFX96 Real-Time PCR system (Bio-Rad, Hercules, CA, USA). The *CaUBI-3* gene was used as an internal control [58]. Relative gene expression levels were calculated using the 2^−ΔΔCt^ method based on three independent biological replicates [59]. Each biological replicate consisted of pooled tissues collected from at least five independent individual plants to ensure biological independence. For floral tissues (e.g., pistils and stamens), each biological replicate was additionally composed of at least 100 individual organs to minimize sampling variation. Primer sequences used for qRT-PCR are listed in Table S5.

### 4.8. GUS Staining

For promoter–GUS reporter construction, a genomic fragment (~2.0–3.0 kb) upstream of the ATG start codon of *CaBBM* was amplified. The resulting fragment was cloned into the pCAMBIA1300 vector to generate a fusion with the β-glucuronidase (GUS) reporter gene. Primer sequences used for vector construction are provided in Table S5. The recombinant constructs were introduced into *Arabidopsis* using the floral dip method [60]. Multiple independent stable T_2_ positive lines were selected for GUS staining analysis. Various tissues from transgenic plants were collected and subjected to histochemical staining using a GUS staining kit (Coolaber, Beijing, China) to evaluate promoter activity.

### 4.9. Subcellular Localization Assay

The full-length coding sequence (CDS) of *CaBBM* was amplified and subsequently cloned into the pFGC5941 vector to generate a GFP-CaBBM fusion expression construct. The primers used for vector construction are listed in Table S5. Transient transformation of tobacco leaves was performed following the method described by Pan et al. [61]. Fluorescence signals were then examined using confocal laser scanning microscopy (Nikon, A1-SHS, Tokyo, Japan).

### 4.10. Generation of Heterologous Overexpression Line in Arabidopsis

The CDS of *CaBBM* was cloned into the pFGC1008 vector to generate a *35S::CaBBM* overexpression construct. The primers used for vector construction are listed in Table S5. *Arabidopsis* Col-0 plants were transformed using the floral dip method. Overexpression levels of transgenic lines were quantified by qRT-PCR. Pollen viability was assessed using Alexander’s staining method [62].

### 4.11. Yeast Two-Hybrid (Y2H) Assay

The Y2H assay was performed using the Matchmaker Gold Yeast Two-Hybrid System (Clontech, Mountain View, CA, USA) following the manufacturer’s instructions. The complete CDS of *CaBBM* was inserted into the pGBKT7 vector. *Saccharomyces cerevisiae* Y2H Gold cells were simultaneously transformed with pGADT7 and pGBKT7-CaBBM and initially grown on SD/-Trp/-Leu (SD/-2) medium for selection. The potential transcriptional activation was then assessed on SD/-His/-Ade/-Trp/-Leu medium supplemented with X-α-Gal (SD/-4). Primer sequences used for vector construction are provided in Table S5.

In the yeast two-hybrid screening, pGBKT7-CaBBM was employed as the bait to screen against the pre-established chili yeast library [57]. A series of 3-amino-1,2,4-triazole (3-AT) concentrations (0, 10, 20, 30, 60, and 80 mM) were evaluated to determine the minimal concentration that effectively suppresses CaBBM self-activation, which was then applied in yeast library screening to prevent false-positive interactions.

## 5. Conclusions

In summary, based on the recently released gap-free T2T genome assembly of pepper, we generated an updated and high-confidence annotation of the AP2/ERF transcription factor family, identifying 155 members and comprehensively characterizing their phylogenetic relationships, gene structures, conserved motifs, chromosomal distribution, collinearity patterns, *cis*-regulatory elements, and expression profiles. We further performed a preliminary functional analysis of *CaBBM*, a representative gene of the AP2 subfamily. *CaBBM* was preferentially expressed in stamens, and its heterologous overexpression in *Arabidopsis* markedly affected root growth, leaf development, and pollen production. In addition, candidate interacting proteins of CaBBM were identified. Together, these findings establish a comprehensive framework for understanding AP2/ERF transcription factors in pepper, and provide theoretical support for future studies on developmental regulation, reproductive biology, and molecular breeding in this species.

## Figures and Tables

**Table 1:** List of the genes encoding CaBBM potential interactors obtained by screening the yeast cDNA library.

Gene ID	Descriptions
CaT2T02g02440	vacuolar protein sorting-associated protein 28 homolog 2
CaT2T08g03186	heavy metal-associated isoprenylated plant protein 26
CaT2T11g01069	histone H2A
CaT2T03g00621	4-coumarate–CoA ligase 1
CaT2T01g04419	60S ribosomal protein L37a
CaT2T01g03681	DNA replication licensing factor MCM7
CaT2T06g00388	ERBB-3 BINDING PROTEIN 1
CaT2T06g01517	cytochrome c1-1, heme protein, mitochondrial
CaT2T03g02367	60S ribosomal protein L36-1
CaT2T01g05462	DNA (cytosine-5)-methyltransferase CMT2
CaT2T04g00354	lysophospholipid acyltransferase LPEAT1
CaT2T07g03325	uncharacterized LOC107878871

## Data Availability

All data in this study can be found in the manuscript or the Supplementary Materials.

## Supplementary Materials

The following supporting information can be downloaded at: https://www.mdpi.com/article/10.3390/plants15152266/s1, Figure S1: Schematic representation of the phylogenetic tree, conserved motifs, and conserved domains of 155 putative AP2/ERF family members in *C. annuum*; Figure S2: Multiple sequence alignment of the AP2 domains from 155 putative AP2/ERF family members in *C. annuum*; Figure S3: *Cis*-elements in the 2 kb promoter regions of AP2/ERF genes in *C. annuum*. Different colored rectangles represent distinct *cis*-elements, with their positions indicated relative to their location within the promoters; Figure S4: Expression profiles of ERF/DREB subfamily genes under cold (A) and heat (B) stress. In the heatmap, red represents high expression levels, while blue represents low expression levels; Figure S5: Expression profiles of ERF/DREB subfamily genes under osmotic (A) and salt (B) stress. In the heatmap, red represents high expression levels, while blue represents low expression levels; Figure S6: Screening of *CaBBM* transgenic plants. (A) Positive transgenic plants were identified through PCR analysis. (B) Relative expression detection of T_1_ generation *CaBBM* heterologously expressing in *Arabidopsis* lines. Values represent mean ± SD of three biological replicates; Figure S7: Effects of *CaBBM* overexpression on floral organs, siliqua size, and pollen viability. (A) Morphology of floral organs and siliqua in WT and *CaBBM*-OE plants. Bars, 1 mm. (B) Alexander staining of pollen from WT and *CaBBM*-OE plants. Bars, 100 μm. (C–E) Statistical results of mature pollen vitality (C), pollen diameter (D), and pollen area (E) (n ≥ 3). The data represents mean ± standard error. Data are presented as mean ± SE. Each replicate included at least 250 pollen grains. Statistical significance was assessed using one-way ANOVA and Dunnett’s multiple comparison tests; ns indicates no significant difference; Figure S8: Screening of CaBBM candidate interacting proteins. (A) Determine the minimum concentration of 3-AT required to suppress CaBBM self-activation. A concentration of 10 mM 3-AT completely inhibited CaBBM self-activation. (B) Positive yeast colonies (blue) obtained from the yeast two-hybrid library screening. (C) KEGG pathway enrichment analysis of the candidate CaBBM-interacting proteins; Table S1: Physicochemical properties of AP2/ERF family gene in *C. annuum*; Table S2: Genes used to construct the phylogenetic tree; Table S3: AP2/ERF Gene pairs exhibited Ka/Ks in *C. annuum*; Table S4: List of the genes encoding CaBBM potential interactors obtained by screening yeast the cDNA library; Table S5: Primers used for qRT-PCR and vector construction; Table S6: Comparison of AP2/ERF family members identified in the T2T genome of the pepper doubled haploid line ‘G1-36576’ and the CM334 v2.0 genome; Table S7: Previously reported AP2/ERF genes excluded from the current T2T-based annotation after conserved domain validation.
