# Supplementary material for: Genome-Wide Identification of AP2/ERF Transcription Factors in Capsicum annuum and Preliminary Functional Analysis of CaBBM
Source: Plants (Basel). 2026 Jul 24;15(15):2266. doi: 10.3390/plants15152266 (PMC13468470; doi:10.3390/plants15152266)
Supplement: Supplementary file 1 [file plants-15-02266-s001.zip › Supplementary Figures.pdf]

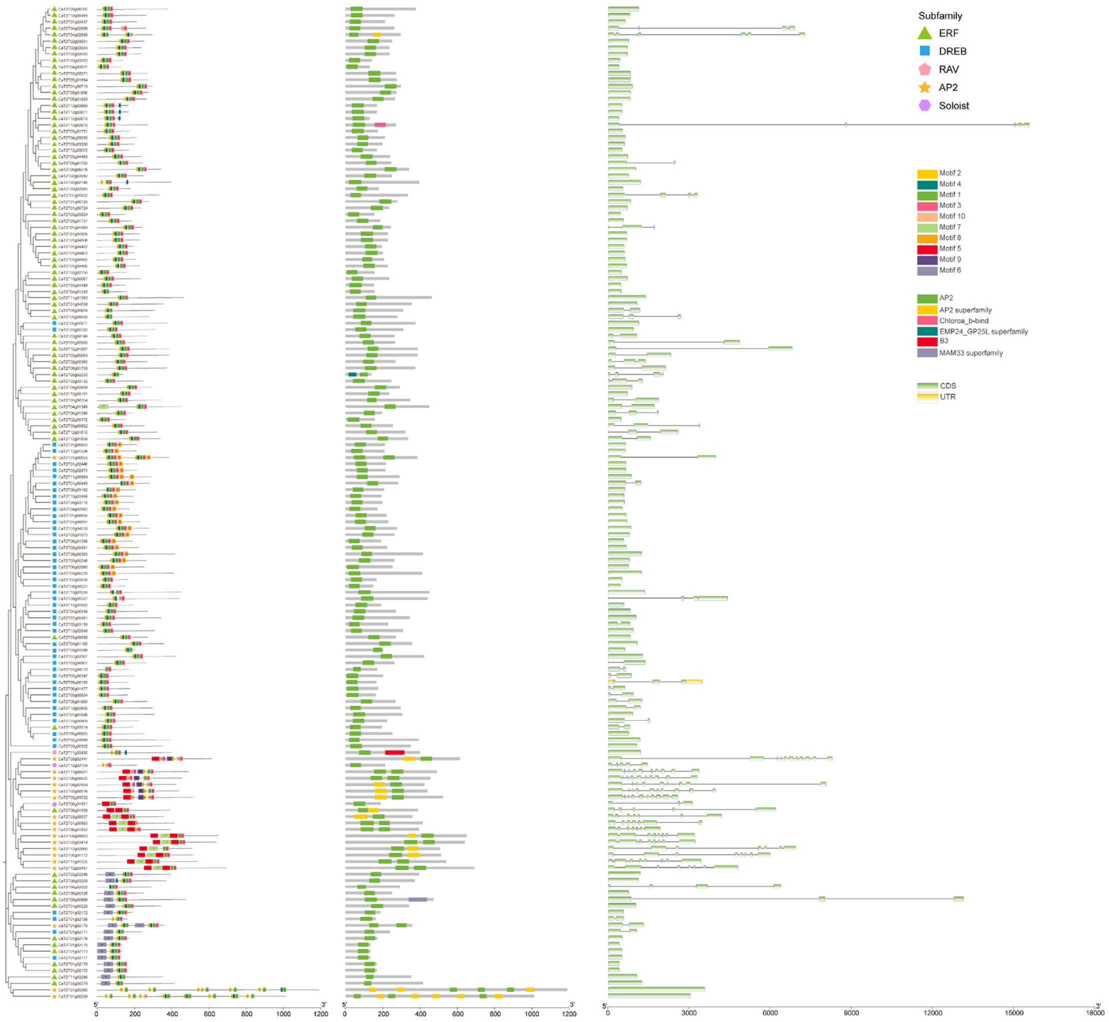

Figure S1. Schematic representation of the phylogenetic tree, conserved motifs, and conserved domains of 155 putative AP2/ERF family members in *C. annuum*.

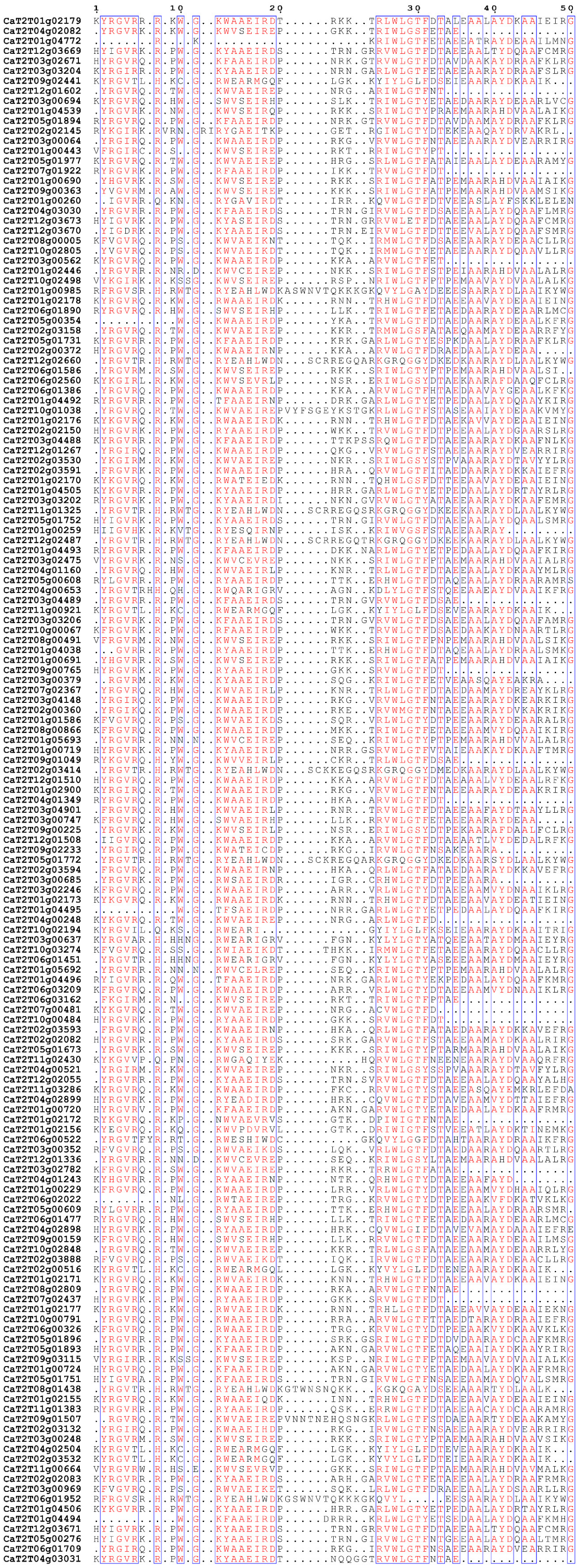

Figure S2. Multiple sequence alignment of the AP2 domains from 155 putative AP2/ERF family members in *C. annuum*.

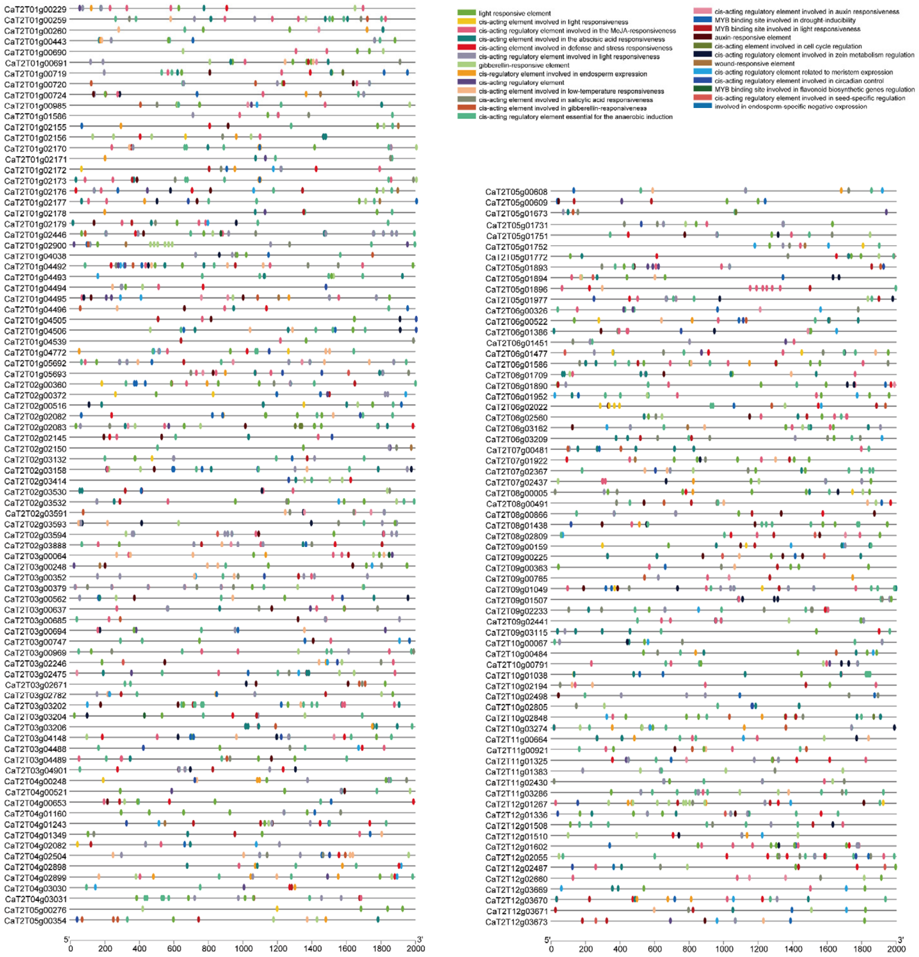

Figure S3. *Cis*-elements in the 2 kb promoter regions of AP2/ERF genes in *C. annuum*. Different colored rectangles represent distinct *cis*-elements, with their positions indicated relative to their location within the promoters.

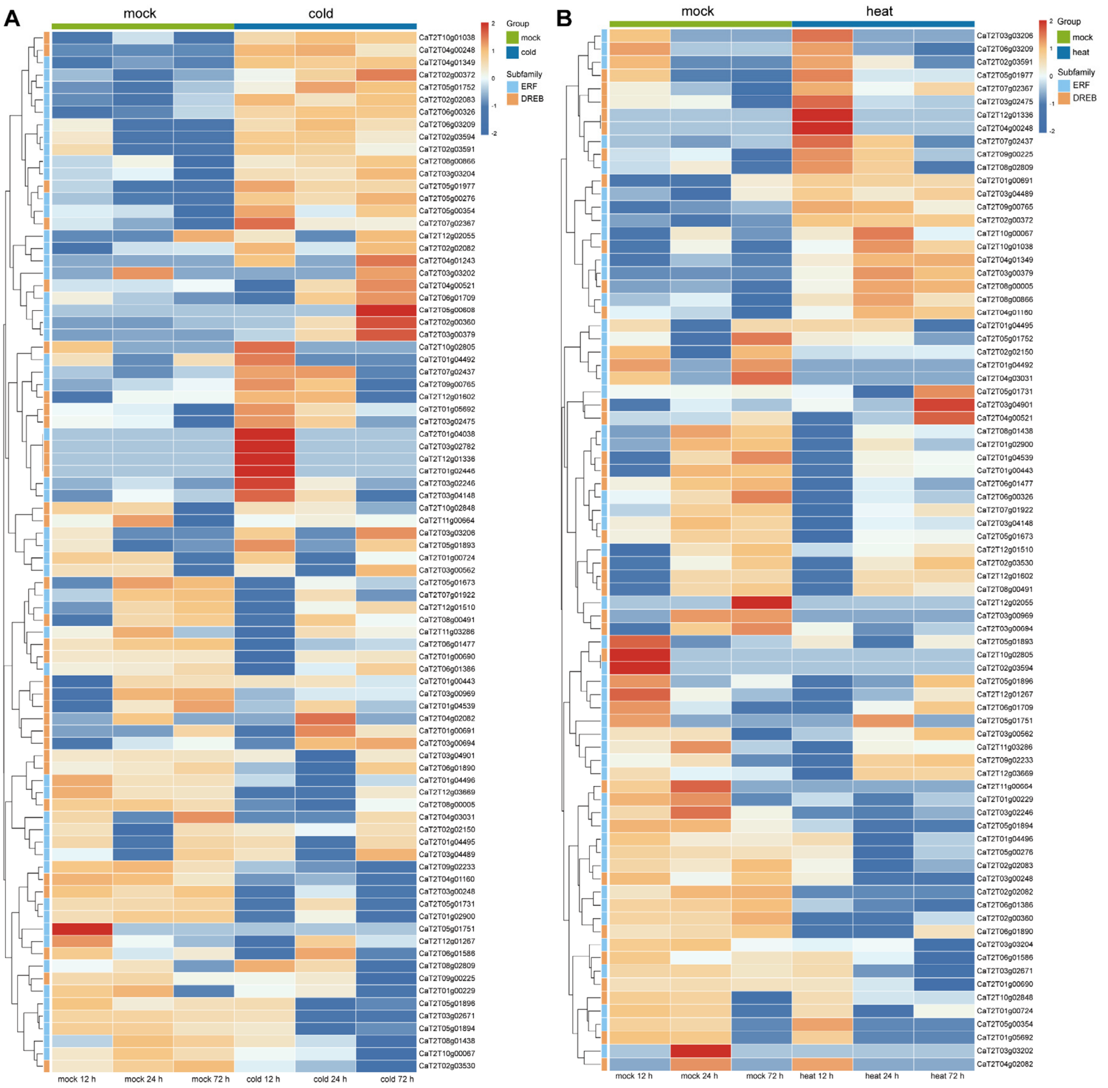

Figure S4. Expression profiles of ERF/DREB subfamily genes under cold (A) and heat (B) stress. In the heatmap, red represents high expression levels, while blue represents low expression levels.

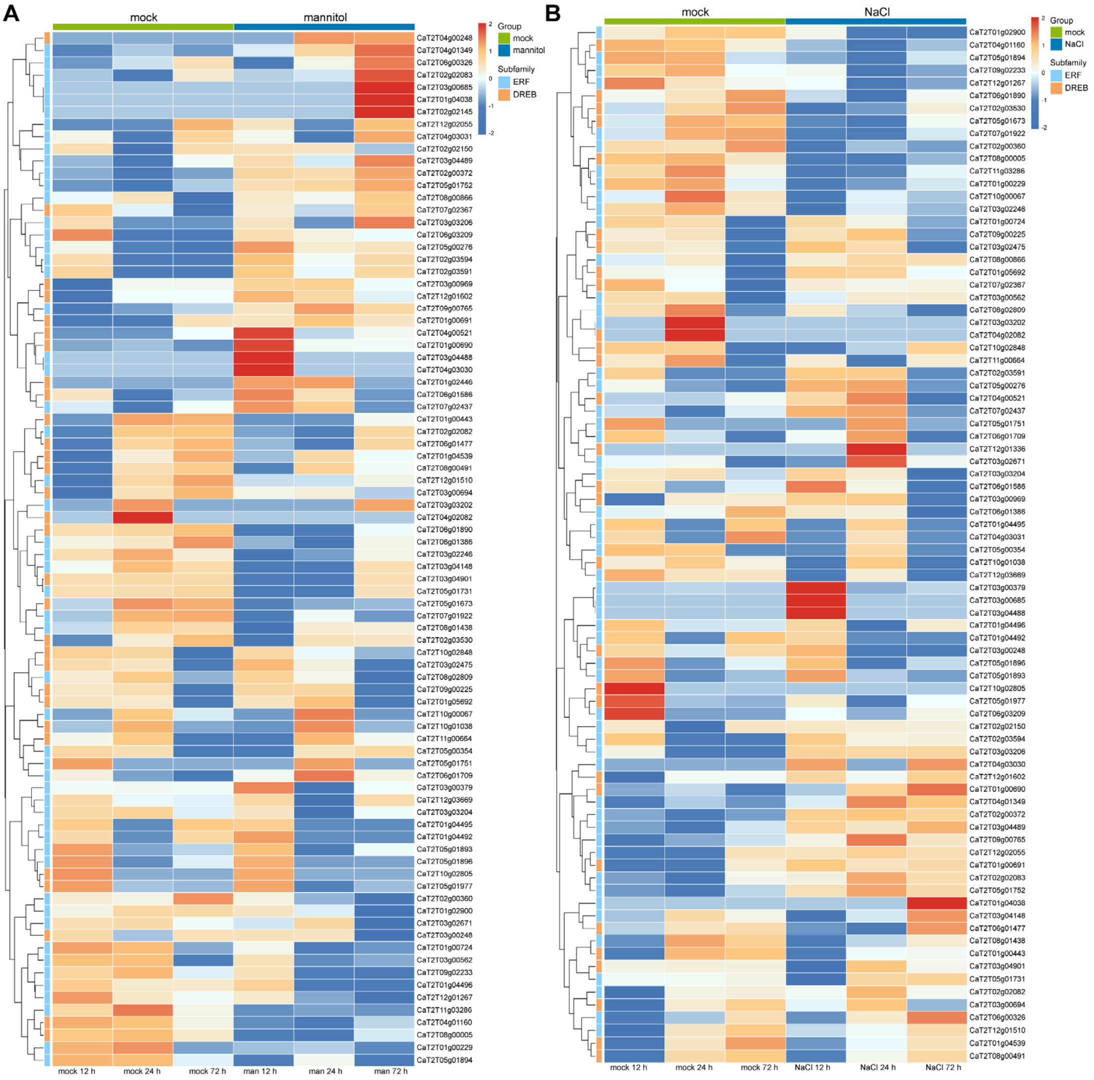

Figure S5. Expression profiles of ERF/DREB subfamily genes under osmotic (A) and salt (B) stress. In the heatmap, red represents high expression levels, while blue represents low expression levels.

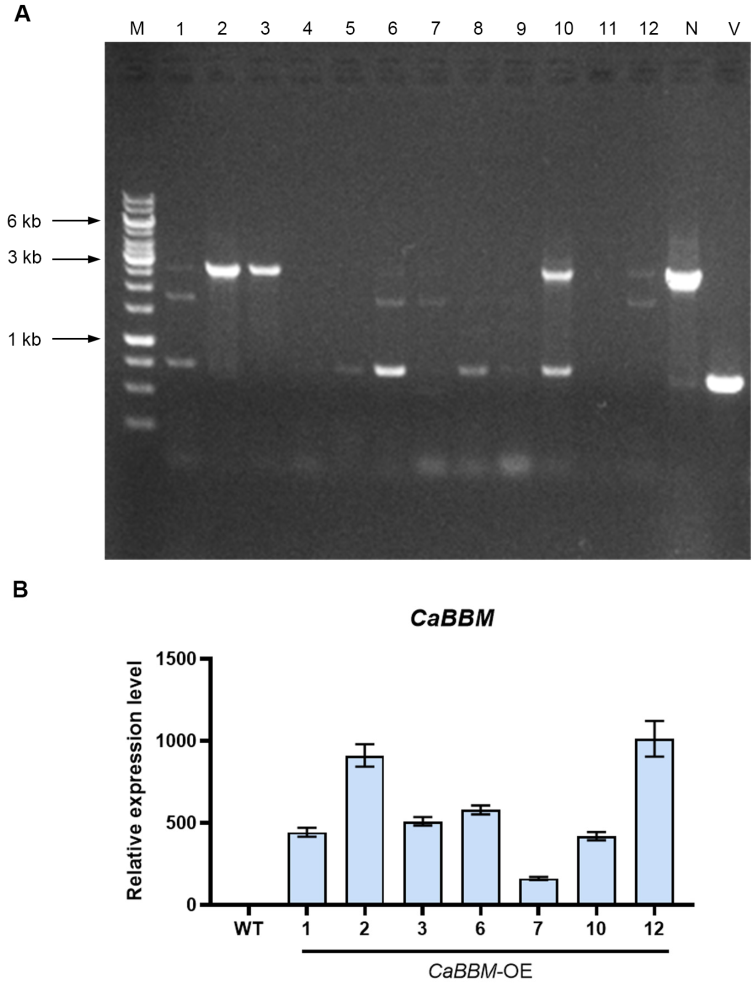

Figure S6. Screening of *CaBBM* transgenic plants. (A) Positive transgenic plants were identified through PCR analysis. (B) Relative expression detection of T_1 generation *CaBBM* heterologously expressing in *Arabidopsis* lines. Values represent mean ± SD of three biological replicates.

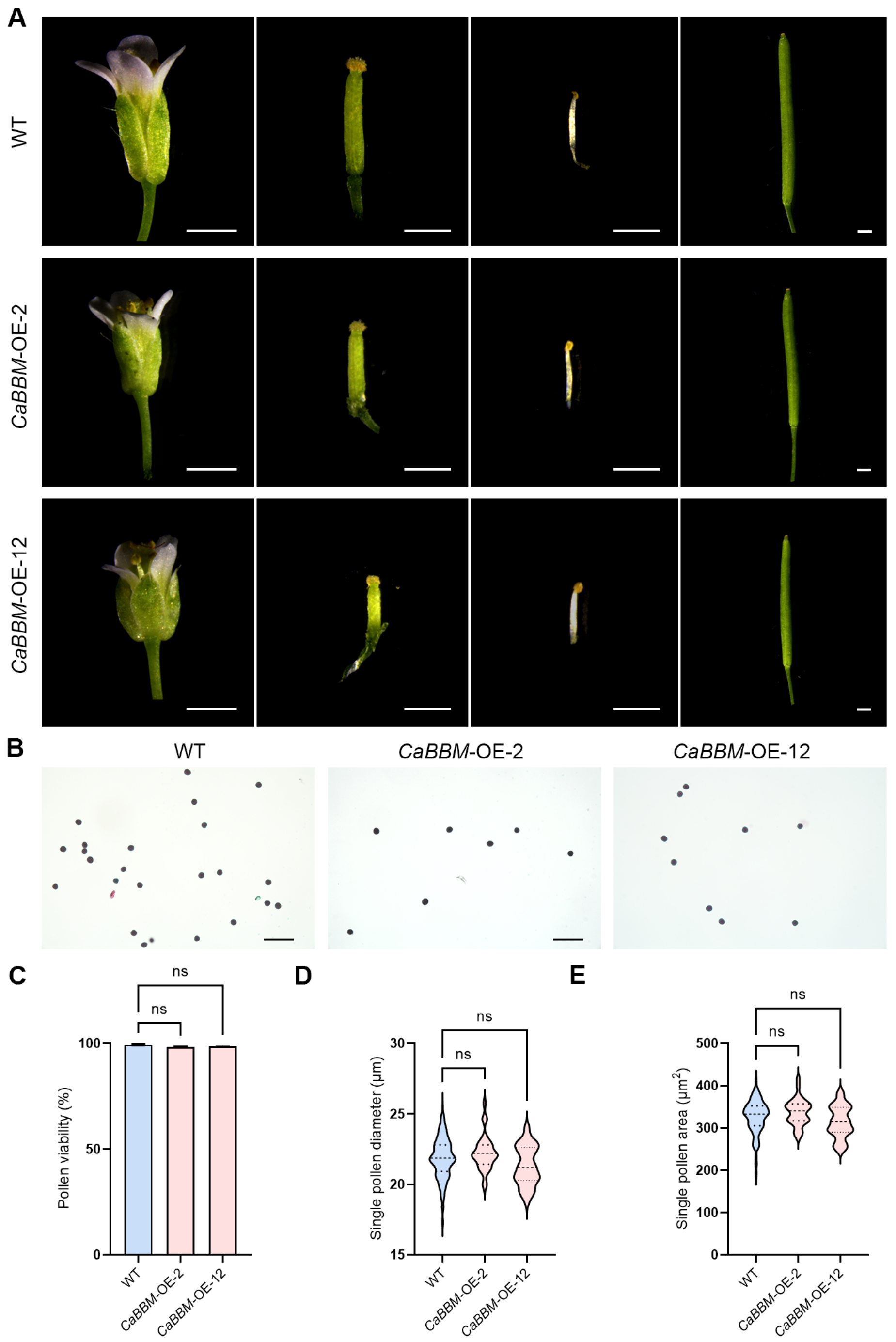

Figure S7. Effects of *CaBBM* overexpression on floral organs, siliqua size, and pollen viability. (A) Morphology of floral organs and siliqua in WT and *CaBBM*-OE plants. Bars, 1 mm. (B) Alexander staining of pollen from WT and *CaBBM*-OE plants. Bars, 100 μm. (C-E) Statistical results of mature pollen vitality (C), pollen diameter (D), and pollen area (E) (n ≥ 3). The data represents mean ± standard error. Data are presented as mean ± SE. Each replicate included at least 250 pollen grains. Statistical significance was assessed using one-way ANOVA and Dunnett's multiple comparison tests; ns indicates no significant difference.

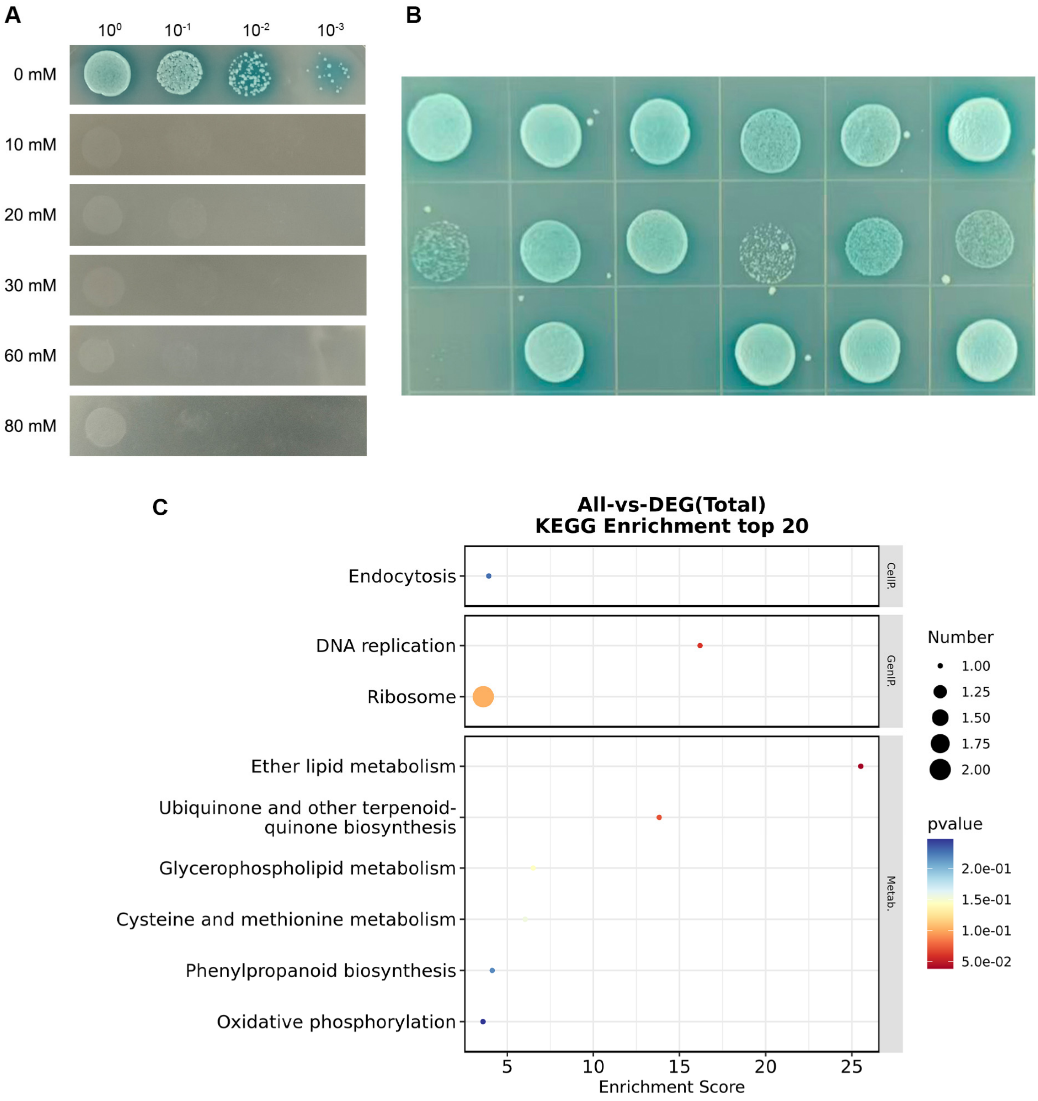

Figure S8. Screening of CaBBM candidate interacting proteins. (A) Determine the minimum concentration of 3-AT required to suppress CaBBM self-activation. A concentration of 10 mM 3-AT completely inhibited CaBBM self-activation. (B) Positive yeast colonies (blue) obtained from the yeast two-hybrid library screening. (C) KEGG pathway enrichment analysis of the candidate CaBBM-interacting proteins.
